# Aberrant Hepatic Methionine Metabolism and Gene Methylation in the Pathogenesis and Treatment of Alcoholic Steatohepatitis

**DOI:** 10.1155/2012/959746

**Published:** 2011-09-06

**Authors:** Charles H. Halsted, Valentina Medici

**Affiliations:** Department of Internal Medicine, University of California, Davis, 451 E. Health Sciences Drive, Room 6323, Davis, CA 95616, USA

## Abstract

The pathogenesis of alcoholic steatohepatitis (ASH) involves ethanol-induced aberrations in hepatic methionine metabolism that decrease levels of S-adenosylmethionine (SAM), a compound which regulates the synthesis of the antioxidant glutathione and is the principal methyl donor in the epigenetic regulation of genes relevant to liver injury. The present paper describes the effects of ethanol on the hepatic methionine cycle, followed by evidence for the central role of reduced SAM in the pathogenesis of ASH according to clinical data and experiments in ethanol-fed animals and in cell models. The efficacy of supplemental SAM in the prevention of ASH in animal models and in the clinical treatment of ASH will be discussed.

## 1. Introduction

Alcoholic steatohepatitis (ASH) represents the intermediate stage of liver injury in the progression of steatosis, inflammation, and hepatocellular apoptosis and necrosis that occurs in the development of alcoholic cirrhosis. This paper will address the role of ethanol-induced aberrant hepatic methionine metabolism in the pathogenesis of ASH, in particular through its epigenetic effects on the expressions of genes relevant to alcoholic liver injury. Initially, we will discuss the normal hepatic methionine metabolic cycle, including its interactions with folate, vitamin B12, and vitamin B6, and its relevance to the epigenetic regulation of gene expression. This will be followed by experimental evidence for multiple effects of ethanol on the methionine metabolic cycle that lead to liver injury through altered methylation and expressions of genes relevant to steatosis, apoptosis, and oxidative stress. We will summarize data from experimental and clinical studies that support these concepts.

## 2. Normal Hepatic Methionine Metabolism

**Figure 1:**
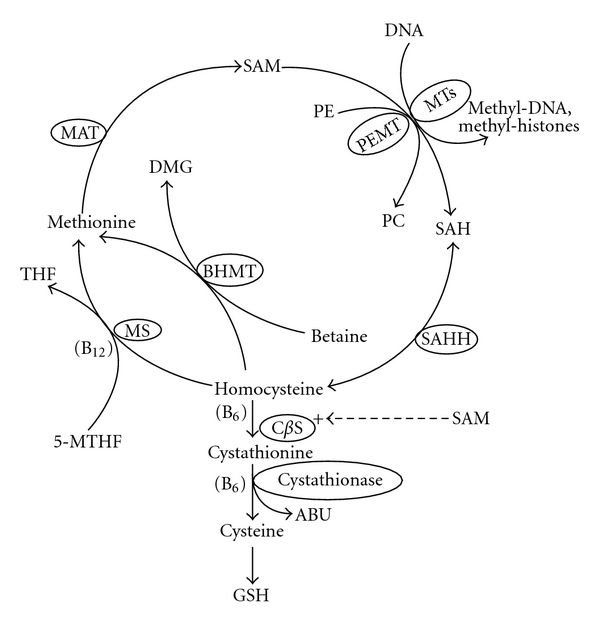
Normal hepatic methionine metabolism. 5-MTHF: 5-methyltetrahydrofolate; THF: tetrahydrofolate; MS: methionine synthase; BHMT: betaine hydroxy methyltransferase; DMG: dimethylglycine; MAT: methionine adenosyl transferase; SAM: S-adenosylmethionine; MT: methyltransferase; PE: phosphatidylethanolamine; PEMT: phosphatidylethanolamine methyltransferase; PC: phosphatidylcholine; SAH: S-adenosylhomocysteine; SAHH: SAH hydrolase; C*β*S: cystathionine beta synthase; ABU: *α*-aminobutyrate; GSH: glutathione.

Hepatic methionine metabolism can be visualized in two parts, the transmethylation cycle for production of S-adenosylmethionine (SAM) and its metabolism to homocysteine and the transsulfuration pathway for the reduction of homocysteine for the synthesis of glutathione (GSH) (Figure 1). Endogenous 5-methyltetrahydrofolate (5-MTHF) that is derived from dietary folate is substrate for the initial vitamin-B12-dependent reaction of methionine synthase (MS) that converts homocysteine to methionine. The additional pathway of betaine homocysteine methyltransferase (BHMT) uses the choline product betaine as substrate with homocysteine for methionine synthesis and is considered a salvage pathway when MS is compromised by toxins including ethanol. Methionine is metabolized to SAM by methionine adenosyltransferase (MAT), which is a product of the MAT1A gene in adult liver. SAM is utilized at a rate of 6–8 grams per day [1], primarily by different methyltransferase reactions that include phosphatidylethanolamine transferase (PEMT) to yield phosphatidylcholine (PC) and many others that methylate gene-specific DNA and various histone residues. In addition, SAM levels are regulated by the activity of glycine-n-methyltransferase (GNMT) with sarcosine as its end product (not shown in Figure 1). While SAM is the sole substrate for methyltransferase reactions, its product S-adenosylhomocysteine (SAH) is the principal inhibitor of the same reactions. Consequently, the SAM-to-SAH ratio is used conveniently as an index of methylation capacity since the Km and Ki for most of these reactions are in similar range [2]. SAH is hydrolyzed to homocysteine by SAH hydrolase (SAHH), which is a reversible enzyme depending upon the abundance of each product. The initial reaction in the transsulfuration pathway is facilitated by SAM and involves the reduction of homocysteine through the cystathionine beta synthase (C*β*S) reaction to cystathionine [3]. Both C*β*S and the subsequent cystathionase reaction require vitamin B6 as cofactor. Therefore, it may be predicted that conditions that limit the production of SAM and the availability of vitamin B6 reduce the production of GSH and its antioxidant properties. Summarizing the key points, homocysteine is regulated by three enzymes, MS, SAHH, and C*β*S, each of which can be compromised to result in homocysteine elevation which is involved in the pathogenesis of ASH. The metabolite SAM plays a key role in regulating gene methylation and expression and a second one by regulating the production of the principal antioxidant GSH. 

## 3. Effects of Ethanol Exposure on Hepatic Methionine Metabolism

There is abundant and accumulating evidence that experimental ethanol exposure and chronic alcoholism influence the expressions and activities of several enzymes in the hepatic methionine cycle. For example, transcript expressions of MS, MAT1A, and C*β*S were decreased in liver biopsies from ASH patients [4], while decreased expressions and activities of MS, MAT1A, and SAHH, but increased GNMT, were found in chronic ethanol-fed micropigs with histologically proven ASH [5]. Reduced activities of MS with compensatory increase in BHMT expressions and activities were also found in ethanol-fed rats [6–8]. Since MAT1A is susceptible to oxidation and nitrosylation of its cysteine 121 residue, its reduced activity in ASH may be attributed to the generation of reactive oxygen species (ROS) that are produced by the ethanol metabolizing enzyme cytochrome p4502E1 (CYP2E1) [9, 10]. Thus, since SAM regulates the generation of GSH through its facilitating effect on C*β*S [3], the reduced antioxidant potential caused by reduced GSH would predictably decrease the effectiveness of MAT1A to generate SAM. These relationships were supported by in vitro studies in chemically treated primary hepatocytes in which elevation of prooxidant CYP2E1 was associated with reduced SAM levels [11]. Also, studies in ethanol-fed baboons and micropigs showed close correlations between liver levels of GSH and SAM [12, 13].

## 4. Effects of Aberrant Methionine Metabolism on the Pathogenesis of ASH

The conventional understanding of the pathogenesis of ASH includes a series of events that are triggered by ethanol-induced transport of enterotoxic lipopolysaccharide (LPS) from the intestine to hepatic Kupffer cells that are in turn induced to release cytokines such as tumor necrosis factor alpha (TNF*α*) which initiates pathways of hepatocyte apoptosis, inflammation, and necrosis. These processes are facilitated by the effect of the ethanol metabolizing hepatocyte enzyme CYP2E1 which is a powerful producer of ROS [14]. In addition, the metabolism of ethanol by alcohol to acetaldehyde alters the NADH/NAD redox potential which promotes steatosis through its effects on fatty acid oxidation in the liver [15]. Steatosis, which is an early sign in development of ASH, represents the combination of increased hepatic lipogenesis and decreased fatty acid oxidation and reduced triglyceride export. Many of these avenues of injury have been linked to ethanol-induced aberrant methionine metabolism in experimental animal models, as described below. 

Studies that used the model of the intragastric ethanol-fed C57BL6 mouse linked enhanced lipogenesis and apoptosis to hyperhomocysteinemia, which is known to trigger endoplasmic reticulum (ER) stress pathways that include upregulation of the chaperone glucose-regulated protein (GRP78), the transcription factor sterol response element binding protein (SREBP1-c) and its downstream lipogenesis genes, and the growth arrest and DNA damage (GADD153) pathway for apoptosis. Since the histopathology of steatosis and apoptosis and these gene activations were prevented by concomitant provision of betaine which lowered homocysteine through the BHMT salvage pathway (Figure 1), the authors concluded that there is a specific role for ethanol-induced hyperhomocysteinemia in the pathogenesis of ASH by activation of ER stress pathways [16]. More recent studies in the ethanol-fed rat model of ASH associated apoptosis with increased liver SAH and steatosis with reduced activity of PEMT which regulates triglyceride export from the liver [17–19]. Others associated apoptosis in ethanol-fed mice with increased levels of SAH which sensitized isolated primary hepatocytes to the effects of TNF*α* [20], while a later study in the same model associated this effect with reduced mitochondrial SAM (mSAM) caused by competitive inhibition of its mitochondrial transport by elevated SAH [21]. The original finding of a mitochondrial transporter for cytoplasmic SAM that is inhibited by SAH [22] was complemented by discovery of a mitochondrial GSH transporter that is inactivated by ethanol but is sustained by coadministration of SAM [23]. A role for mSAM in prevention of the prooxidant effects of ethanol was supported by intervention studies that demonstrated a protective effect of SAM against decreased mitochondrial respiration [24] and increased levels of mitochondrial superoxide and inducible nitric oxide synthase (iNOS) [25]. SAM may also play a role in prevention of hepatocellular carcinoma (HCC) in ALD by inhibition of angiogenesis genes [26].

## 5. Role of Altered Gene Methylation Capacity in the Pathogenesis of ASH

The pathogenesis of ASH includes activations of many genes involved in liver injury, each of which are potentially and epigenetically regulated by alterations in hepatic levels of SAM and SAH and their methylation capacity ratio. Our laboratory explored these relationships in two ethanol-fed animal models, the micropig and the genetically altered mouse. An initial study demonstrated the suitability of the micropig for study of the pathogenesis of ASH, in which its characteristic histopathology was induced by feeding ethanol at 40% of kcal over 12 months [27] together with progressive increase in serum homocysteine levels and reduced gene methylation capacity as shown by decrease in the liver SAM and the SAM-to-SAH ratio [28]. Subsequently, we included folate deficiency in the dietary regimen in order to accentuate changes in methionine metabolism through reduction of the initial methyl donor 5-MTHF (Figure 1), with the additional rationale of the well-known association of folate deficiency with chronic alcoholism [29, 30]. In contrast to the findings in the previous study [27] only 3 months were required for the development of the histopathology of ASH, while increases in timed serum homocysteine levels and lowering of the hepatic SAM-to-SAH ratio were the greatest in the group fed the combined folate-deficient and ethanol diet compared to groups fed either diet alone [12]. Using liver samples from the same groups of micropigs, we found progressive increase in transcript and protein levels of genes relevant to steatosis and liver injury from micropigs fed control, folate-deficient, ethanol, and ethanol with folate-deficient diets, together with increased expressions of CYP2E1 and ER stress pathway genes for the marker GRP 78, lipogenesis that included SREBP 1-c and fatty acid synthase (FAS), and apoptosis including GADD 153 [31]. The finding that these gene expressions correlated with changes in the SAM-to-SAH ratio supported the relationship of the pathogenesis of ASH to ethanol-induced reduction of gene methylation capacity. This concept was supported by subsequent studies in which the coadministration of SAM sustained normal histology while preventing changes in the SAM-to-SAH ratio, and activations of the same ER stress genes for lipogenesis and apoptosis [32], as well as genes relevant to oxidative liver injury including CYP2E1, nicotinamide adenine dinucleotide phosphate oxidase (NOX1), and inducible nitric oxide synthase (iNOS) [33].

## 6. ASH Is Mediated through the Epigenetic Effects of Ethanol-Induced Altered Methionine Metabolism

Epigenetics refers to the effects of external factors on the expressions of genes that are unrelated to changes in their DNA sequences. Gene expression is regulated at the levels of both DNA and its histone infrastructures that exist within nuclear chromatin. In the context of the present paper, such changes can be attributed to the regulatory effects of increased or decreased methylation on DNA and histones, since folate contains the parent methyl group and SAM is the principal methyl donor in all methyltransferase reactions (Figure 1). As described in recent reviews, the effect of chronic ethanol exposure on the activation or suppression of selected ASH-related genes can be ascribed to different methylation effects at histone 3 lysine (K) residues, whereby gene expression is enhanced by methylation at H3meK4 but is silenced by methylation at H3meK9 [34, 35]. Furthermore, ethanol exposure enhances gene expression by acetylation at H3AcK9[35], whereas sirtuin1, a known histone de-acetylase, is reduced in livers of ethanol-fed mice [36]. Therefore, the effects of ethanol exposure on specific histone methyltransferases, acetylases, and deacetylases may be crucial to the epigenetic control of the expressions of genes relevant to liver injury. Further epigenetic regulation occurs at the level of DNA, where increased methylation of promoter region cytosine residues suppresses gene expression and vice versa. For example, global DNA hypomethylation occurred in the ethanol-fed SAM-deficient rat [37] and MAT1A knockout mouse with enhanced carcinogenesis [38], as well as in our folate-deficient chronic alcoholic micropig model in association with increased DNA oxidation and strand breaks [12]. Others found reduced expression of the DNA methyltransferase DNMT3b that correlated negatively with increased blood alcohol levels in chronic alcoholics together with increased global DNA methylation [39]. 

We explored epigenetic regulation of ASH in a novel model of the C57BL6 C*β*S-deficient mouse fed intragastric ethanol at 37% of kCal for 4 weeks. This model is particularly relevant since, as noted above, the same method was used to induce ASH in C57BL6 wild-type mice with findings of altered methionine metabolism, all of which were corrected by the methyl donor betaine [16]. Since C*β*S heterozygosity is known to increase serum homocysteine levels [40] and ethanol exposure inhibits MS expression and activity [5, 6] we reasoned that both ethanol exposure and genotype would accentuate aberrant methionine metabolism with the greatest effect in the combined group. Our study showed all the histopathological features of severe ASH in the combined ethanol-fed heterozygous group, which also showed the greatest reduction in the SAM-to-SAH ratio and the greatest increase in expressions of the ER stress genes SREBP 1-c, GRP78, and GADD153 [41]. While immunohistological staining of liver slices showed that methylated histone residue H3K9 was specifically reduced in centrilobular regions which were the sites of maximal steatosis in the ethanol-fed heterozygous group, the chromatin immunoprecipitation (ChIP) assay using antibody to H3K9 showed its reduced presence in the promoter regions of the same ER stress genes. Since H3K9 associates with gene suppression, its reduced presence in liver from the combined ethanol-fed heterozygous group is consistent with activation of these genes. Furthermore, the transcript expression of G9a, a histone methyltransferase that is required for methylation of K9 residues, was specifically reduced in both ethanol-fed groups in contrast to other K9 methyltransferases [41]. The mechanism for reduced expression of the histone methyltransferase G9a is unclear, but others have shown effects of ethanol-induced ROS on chromatin remodeling as a mechanism for changes in histone acetylation reactions [42]. Summarizing, these studies provided an epigenetic mechanism for the relationship of ethanol-induced aberrant methionine metabolism on the epigenetic regulation of expression of selected ER stress genes that are relevant to lipogenesis and apoptosis in ASH [41]. 

Using the ChIP assay in a mouse macrophage RAW cell line and in vivo in LPS-treated mice, others showed that SAM inhibited the LPS-induced activations of TNF*α* and iNOS by blocking the binding of H3meK4 to the promoter regions of these genes [43]. Data from two groups suggest epigenetic regulation of proteosome inhibition as a mechanism for enhanced liver injury. In one study in an ethanol-fed rat model, proteosome inhibition was associated with alterations in histone acetylation and methylation [44], whereas methylation of a specific subunit by provision of SAM prevented proteosome degradation in ethanol-exposed hepatocytes [45].

## 7. Clinical Studies

A study of patients with cirrhosis of diverse etiologies including alcoholism found significant elevations in serum homocysteine and cystathionine, which was suggestive of a block in the transsulfuration pathway for homocysteine elimination and production of GSH [46]. Compared to healthy subjects, we found increased serum homocysteine levels in alcoholics with or without ASH in association with elevated serum SAH levels [47]. While others have described elevated homocysteine levels in chronic alcoholics in association with deficiencies of folate and vitamin B6 [48], we found that ASH patients also had marked increase in serum levels of cystathionine, the substrate for vitamin-B6-dependent cystathionase (Figure 1), with comparable reduction in its product *α*-aminobutyrate (ABU). Furthermore, cystathionine levels and the ABU-to-cystathionine ratio correlated with vitamin B6 levels and the ABU-to-cystathionine ratio was a positive predictor of the presence of ASH among alcohol drinkers [47]. 

The growing experimental evidence linking impaired methionine metabolism with the onset and development of alcoholic liver disease prompted several clinical trials that attempted to demonstrate a role for SAM in its treatment. One study showed that 15 days of intravenous SAM at 2 g/day increased red blood cell GSH in 20 patients with ASH [49]. Hepatic GSH levels were low in liver biopsies from 17 patients with ASH or other chronic liver diseases and were normalized by SAM treatment at 1.2 g/day in a 6-month controlled study [50]. A later 2-year multicenter European trial of 123 ASH patients found reduction in mortality or incidence of liver transplant from 30% in the placebo group to 16% in the SAM group, which was significant after exclusion of Childs class C patients from the analysis [51]. A 2006 meta-analysis of 9 studies found insufficient evidence for the effective use of SAM in the treatment of alcoholic liver disease [52]. Whereas the PC derivative polyenylphosphatidylcholine (PPC) was known to restore liver SAM levels in ethanol fed rats [53], there was no benefit of this compound according to liver histopathology in 412 ASH patients who underwent liver biopsies at baseline and after 24 months of treatment [54]. 

We conducted a randomized treatment trial in 26 ASH patients who participated in our baseline study [47] and then received SAM at 1.2 g/d or placebo daily for 24 weeks [55]. All biochemical parameters of liver function improved over time with no differences between the groups. Although serum SAM levels rose over time with treatment, there were no changes in other methionine metabolites in either group. Furthermore, there were no differences between groups in histopathology scores for steatosis, inflammation, or fibrosis in 14 patients who underwent liver biopsies before and after treatment [55]. 

Summarizing, the results from several clinical trials of SAM in treatment of ASH are inconclusive as to its effect. Previous positive studies included patients with other causes of liver disease and showed no comprehensive histological data on the efficacy of SAM. While our study is the first to provide data on potential changes in methionine metabolite levels and documents the lack of differences in histopathology, its limitations include relatively short exposure to SAM and relatively small number of subjects. Unlike studies in animal models that showed the efficacy of SAM in prevention of ASH [13, 32, 33], the use of SAM in the treatment of established liver disease may be compromised by lack of retention of SAM by injured hepatocytes as well as decreased numbers of normally functioning hepatocytes. Also, based on our methionine metabolite findings at baseline [47] and the role of vitamin B6 in the transsulfuration pathway (Figure 1), there may be a requirement for vitamin B6 supplementation in addition to SAM in longer-term treatment of ASH.

## 8. Conclusions

Abundant evidence now exists for the central role of aberrant hepatic methionine metabolism in the pathogenesis of ASH. Ethanol and its ROS products inhibit key enzymes in the methionine cycle, in particular those involved in the synthesis of SAM. Since SAM is the principal methyl donor for histones and DNA and facilitates the production of GSH, its reduction influences the methylation and hence epigenetic regulation of many genes involved in alcoholic liver injury as well as the capacity for antioxidant defense in the pathogenesis of ASH. Whereas SAM supplementation has been proven to prevent ASH and its mechanisms in experimental animal models, clinical trials have failed to demonstrate a definitive effect of SAM in the treatment of established ASH.
